# Whole-genome sequencing reveals rare off-target mutations in CRISPR/Cas9-edited grapevine

**DOI:** 10.1038/s41438-021-00549-4

**Published:** 2021-05-01

**Authors:** Xianhang Wang, Mingxing Tu, Ya Wang, Wuchen Yin, Yu Zhang, Hongsong Wu, Yincong Gu, Zhi Li, Zhumei Xi, Xiping Wang

**Affiliations:** 1 State Key Laboratory of Crop Stress Biology in Arid Areas, College of Horticulture, Northwest A&F University, 712100 Yangling, Shaanxi China; 2 College of Enology, Northwest A&F University, 712100 Yangling, Shaanxi China; 3 Key Laboratory of Horticultural Plant Biology and Germplasm Innovation in Northwest China, Ministry of Agriculture, Northwest A&F University, 712100 Yangling, Shaanxi China; 4 Novogene Technologies Corporation, 100000 Beijing, China; 5 OEbiotech Corporation, 200000 Shanghai, China

**Keywords:** Molecular engineering in plants, Agricultural genetics

## Abstract

The CRISPR (clustered regularly interspaced short palindromic repeats)-associated protein 9 (Cas9) system is a powerful tool for targeted genome editing, with applications that include plant biotechnology and functional genomics research. However, the specificity of Cas9 targeting is poorly investigated in many plant species, including fruit trees. To assess the off-target mutation rate in grapevine (*Vitis vinifera)*, we performed whole-genome sequencing (WGS) of seven Cas9-edited grapevine plants in which one of two genes was targeted by CRISPR/Cas9 and three wild-type (WT) plants. In total, we identified between 202,008 and 272,397 single nucleotide polymorphisms (SNPs) and between 26,391 and 55,414 insertions/deletions (indels) in the seven Cas9-edited grapevine plants compared with the three WT plants. Subsequently, 3272 potential off-target sites were selected for further analysis. Only one off-target indel mutation was identified from the WGS data and validated by Sanger sequencing. In addition, we found 243 newly generated off-target sites caused by genetic variants between the Thompson Seedless cultivar and the grape reference genome (PN40024) but no true off-target mutations. In conclusion, we observed high specificity of CRISPR/Cas9 for genome editing of grapevine.

## Introduction

Sequence-specific nucleases (SSNs) have been widely used for genome editing, and widely used genome editing tools include ZFNs (zinc finger nucleases)^1^, TALENs (transcription activator-like effector nucleases)^2^ and, more recently, the CRISPR (clustered regularly interspaced short palindromic repeats)-associated protein 9 (Cas9) system^3^. Compared with ZFNs and TALENs, the CRISPR/Cas9 system is relatively easy to deploy^4,5^ and has facilitated targeted gene editing and functional genomics research in plants^6–14^. The CRISPR/Cas9 system uses an RNA-protein complex consisting of two essential components: a Cas9 effector protein and a single guide RNA (sgRNA) containing a targeting sequence of ~20 nucleotide (nt)^3,15^. Once the RNA-protein complex has been introduced into a cell, the sgRNA recognizes a complementary target DNA site with a canonical NGG and noncanonical NGA or NAG protospacer adjacent motif (PAM) sequence and guides the Cas9 endonuclease to induce DNA double-stranded breaks (DSBs)^13,16^. Cells can only ensure normal activity by repairing DSBs by either nonhomologous end-joining (NHEJ) or homology-directed repair (HDR)^3^. This DSB repair process often leads to on-target and off-target mutations^13^. The latter occurs due to the ability of the sgRNA to recognize genomic sites with a few nucleotide mismatches. However, the binding and cutting efficiencies are lower when the sgRNA recognizes DNA with mismatches^17^.

Early studies reported high frequencies of Cas9-induced off-target mutations in human cells^18,19^, and there is considerable interest in understanding the factors that dictate the number and positions of off-target mutations^13,19^. At present, methods such as targeted sequencing, exome sequencing, WGS (whole genome sequencing), BLESS (direct in situ breaks labeling, enrichment on streptavidin, and next-generation sequencing), GUIDE-seq (genome-wide, unbiased identification of DSBs enabled by sequencing), LAM-HTGTS (linear amplification-mediated high-throughput genome-wide translocation sequencing) and Digenome-seq (in vitro Cas9-digested whole genome sequencing) are used for detecting off-target mutations^20^. Of these, targeted sequencing and WGS are currently widely used in plant off-target analysis. Targeted sequencing (amplification and Sanger sequencing) is technically less complex, rapid, and widely available^12,20^. However, this method can only be used to detect a small number of potential off-target sites and is relatively expensive and time-consuming when a larger number of potential off-target sites are screened^20^. By contrast, WGS can be used for comprehensive off-target mutation analysis to reveal variants such as SNPs (single-nucleotide polymorphisms), indels (insertions/deletions), and other structural differences. One possible limitation of WGS is that a reference genome is required^20^. WGS has been used to detect off-target mutations in *Arabidopsis thaliana*^16^, rice (*Oryza sativa*)^17,21^, tomato (*Solanum lycopersicum*)^22^, and cotton (*Gossypium hirsutum*)^13^, among other plants.

Previous reports have suggested that off-target mutations resulting from Cas9 editing of plants are rare. For example, an analysis of 14 Cas9-edited cotton plants revealed only 4 true off-target indel mutations when a combination of WGS and Sanger sequencing were used^13^. In rice, no bona fide off-target mutations were found by WGS in the T1 generation of 34 Cas9-edited plants^17^. Finally, a method termed CIRCLE-seq was used to identify genome-wide potential off-target sites and showed that the CRISPR/Cas9 system is highly specific for genome editing in maize (*Zea mays*)^15^. CRISPR/Cas9 has been successfully used to edit the genomes of fruit tree species, such as apple (*Malus* × *domestica* and *Malus prunifolia* (Wild.) Borkh. ‘Seishi’ × *M. pumila*)^23–25^, orange (*Citrus sinensis* (L.) Osbeck)^26,27^, kiwifruit (*Actinidia deliciosa*)^28,29^, and grapevine (*Vitis vinifera*)^12,30–33^. However, the extent of off-target mutations in CRISPR/Cas9-edited fruit trees is still not completely examined and has mainly been investigated using target sequencing^12,31,32^.

In a previous study, we used CRISPR/Cas9 to edit the grapevine genome and obtained 22 edited lines with no off-target mutations detected by target sequencing^12^. To characterize the potential genome-wide off-target rate in greater depth, in this study, we performed a large-scale WGS analysis of wild-type (WT) and 7 CRISPR/Cas9-edited grapevine plants resulting from individually targeting either of two genes. This approach allowed us to investigate the specificity of CRISPR/Cas9 in grapevine genome editing.

## Materials and methods

### Plant materials and culture conditions

Thompson Seedless floral explants used to induce embryogenic calli were collected from the grape germplasm resource orchard at Northwest A and F University, Yangling, Shaanxi, China. Embryogenic calli and pro-embryonal masses (PEMs) were induced as previously described^12^. All materials were cultivated in the dark at 26 °C and transferred to new media (X6) once per month.

### sgRNA design and vector construction

The online CRISPR-P^34^ (http://cbi.hzau.edu.cn/crispr/) and CRISPR RGEN^35^ (http://www.rgenome.net/) tools were used for sgRNA design. Four *VvbZIP36* targets were chosen according to their GC content, location in the gene, and predicted off-target effects. The sequences of the four sgRNAs used for CRISPR/Cas9 editing are reported in Table S1. The extraction of grape genomic DNA and PCR amplification of the target regions were performed as previously described^12^. Gene-specific primers (*VvbZIP36*-Target-F: 5’-ATGGACGATTTGGAAATTG GGG-3’; *VvbZIP36*-Target-R: 5’-TCACACCAAAACTCCATGAG-3’) were designed based on the grape reference genome sequence (EnsemblPlants, http://plants.ensembl.org/index.html) of VIT_18s0122g00500. Four helper plasmids (PYLsgRNA-LacZ-AtU6-1, -AtU6-29, AtU3d, and -AtU3b) and pYLCRISPR/Cas9P35S-N were used to generate the CRISPR/Cas9 construct^36^. The vector construction methods are outlined in previously published protocols^37^. The primers used in vector construction are listed in Table S2.

### Grapevine transformation

Grapevine transformation was performed as previously described^12,38^. To improve the transformation efficiency, PEMs were transferred to fresh X6 medium and precultured for approximately one week. The multitarget editing vector was transferred into *Agrobacterium tumefaciens* strain EHA105 using the freeze-thaw method^39^. *Agrobacterium*-mediated grapevine PEM transformation was performed according to Dhekney et al.^40^ with minor modifications. Briefly, PEMs were incubated with *A. tumefaciens* strain EHA105 (containing the multitarget editing vector) (OD600, 0.4–0.6) for 7 to 10 min, and then cocultured PEMs were transferred to sterile filter paper in a Petri dish containing an additional three layers of sterile filter paper soaked with DM liquid medium (DKW basal salts, 0.3 g/L KNO_3_, 1.0 mg/L nicotinic acid, 2.0 mg/L each of thiamine-HCl and glycine, 1.0 g/L myo-inositol, 30 g/L sucrose, 5.0 mM 6-BA, 2.5 mM NOA and 2.5 mM 2,4-D, pH 5.7). After 3 days of cultivation in the dark at 26 °C, the PEMs were moved to solid DM medium (200 mg/L carbenicillin and cefotaxime, 75 mg/L kanamycin) and cultivated at 26 °C in the dark. After 1 month, the new embryogenic calli were transferred to X6 medium (200 mg/L carbenicillin and cefotaxime, 75 mg/L kanamycin) to induce transgenic SEs (somatic embryos). Late cotyledonous stage SEs were transferred to MS1B medium (MS salts and vitamins, 0.1 g/L myo-inositol, 20.0 g/L sucrose, 1.0 mM 6-BA, and 7.0 g/L agar, pH 5.8) to regenerate transgenic grapevine plants at 26 °C under white fluorescent light. Vector-specific primers (NPTII-F: 5’-AGAGGCTATTCGGCTATGACTG-3’; NPTII-R: 5’-CAAGCTCTTCAGCAATATCACG-3’) were used to identify stable transgenic plants. The potential edited *VvbZIP36* sequence was amplified using gene-specific primers (*VvbZIP36*-Target-F; *VvbZIP36*-Target-R) to detect on-target mutations. The *VvbZIP36*-Target-F primer was used for Sanger sequencing.

### Whole-genome sequencing

Genomic DNA extraction was extracted from young leaves of seven Cas9-edited and three WT plants using a plant genomic DNA extraction kit (Bioteke, Beijing, China) according to the user manual. Approximately 0.5 µg of DNA was collected to construct sequencing libraries. Library construction and sequencing services were provided by Novogene (Beijing, China). The low-quality reads and adapter sequences were filtered out to obtain clean reads, which were mapped to the grape reference genome (PN40024) and Thomson Seedless genomes (http://openprairie.sdstate.edu/vitis_vinifera_sultanina/1) using BWA^41^ (alignment via Burrows-Wheeler transformation, version 0.7.8-r455, parameters: mem -t 5 -M -R). The grape reference genome (PN40024) and annotations were downloaded from the National Center for Biotechnology Information (NCBI) (GCA_000003745.2, https://www.ncbi.nlm.nih.gov/). The SNPs and indels were identified using SAMtools^42^ (Tools for alignments in the SAM format, version 1.9, parameters: mpileup -q 1 -t DP, DV -m 2 -F 0.002 –ugf) and bcftools^43^ (Tools for variant calling and manipulating VCFs and BCFs, version 1.9, parameters: call -vmO v). The resulting sequences were filtered using bcftools (parameters: QUAL > 20, (INFO/DP) > 4, MQ > 30). The raw WGS data can be found in the NCBI Sequence Read Archive (SRA), BioProject ID: PRJNA677617.

### Prediction of potential off-target sites

The eight sgRNAs were aligned to the grape reference genome in the NCBI database (GCA_000003745.2) using Cas-OFFinder^44^ (http://www.rgenome.net/cas-offinder/), allowing up to 5 mismatches to predict potential off-target sites at the whole-genome level, as previously described^13^. According to the type of PAM, the potential off-target sites were divided into three types (NGG, NAG, and NGA). The SNP and indel variations 100 bp upstream and downstream of all potential off-target sites in the seven Cas9-edited and three WT plants were identified, and all mutations in the potential 20 bp off-target sites were inconsistent with the mutations in the three WT plants were selected for further analysis.

### New potential off-target sites caused by genetic variation between WT and the grape reference genome

The SNP and indel variations shared by the three WT plants were identified and considered genomic variants between Thompson Seedless, the cultivar commonly used for transformation, and PN40024, the cultivar used for reference genome sequencing^45^. The mutations (SNPs and indels) shared by all three WT plants were used to “correct” the reference genome^46^ using an in-house Perl script provided by OE Biotech Co., Ltd. (Shanghai, China). The corrected genome sequence was used for the prediction of potential off-target sites using Cas-OFFinder^44^ with the same parameters as described above.

## Results

### Whole-genome sequencing (WGS) of WT and CRISPR/Cas9-edited grapevine plants

**Fig. 1:**
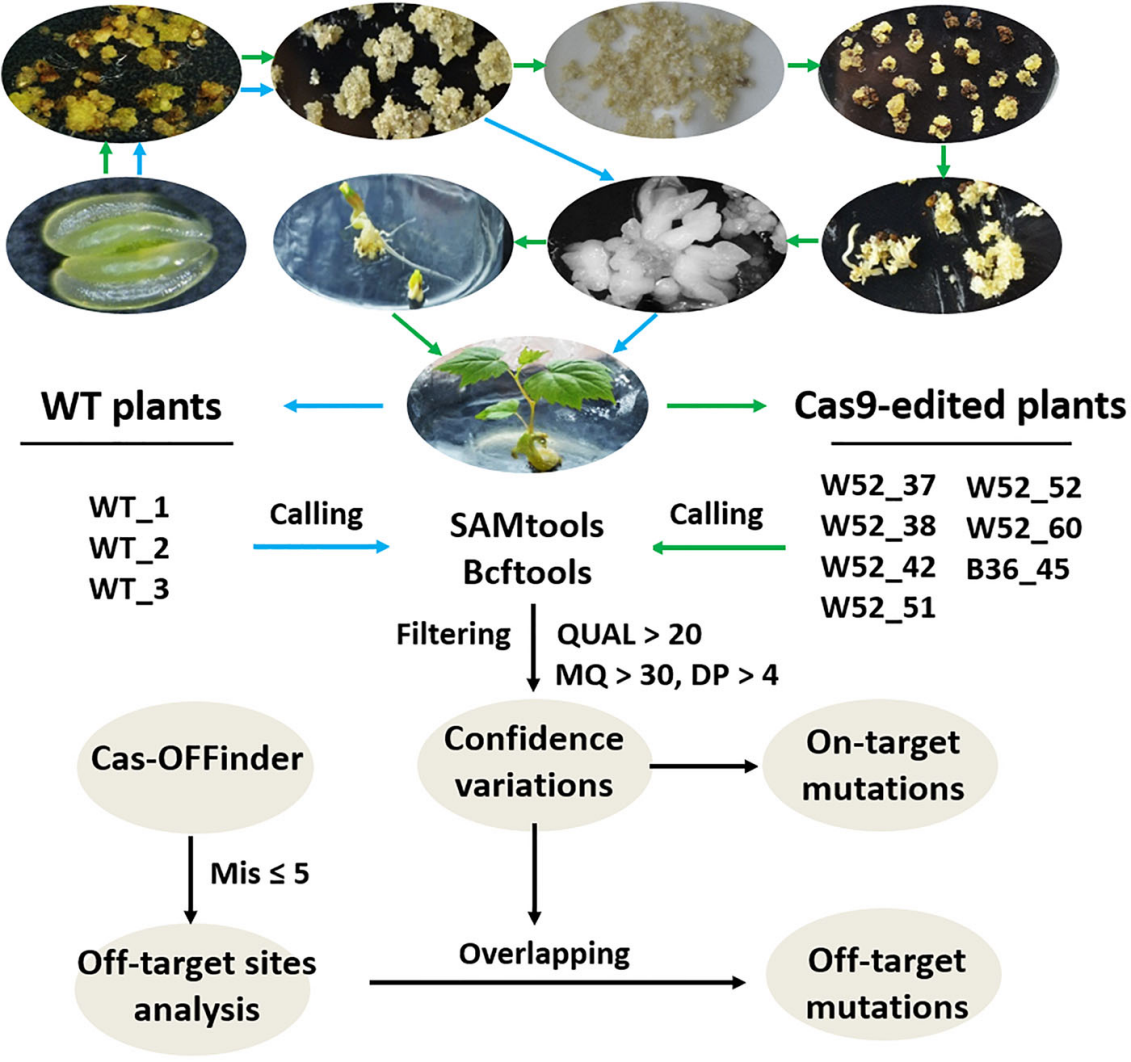
Schematic diagram of on-target and off-target analysis by whole-genome sequencing. Three wild-types (WT) plants and seven Cas9-edited plants (six T0 plants from WRKY52 (W52) editing and one from bZIP36 (B36) editing) were used for whole-genome sequencing. Embryogenic calli were generated from anthers and induced to obtain somatic embryos, which were then used to generate WT plants (blue arrow). *Agrobacterium* was used to infect the pro-embryonal masses (induced from anthers), embryogenic calli and somatic embryos were reinduced, and a transgenic Cas9-induced plant was produced (green arrow). All ten plants regenerated from tissue culture were used for whole-genome sequencing analysis. The bioinformatics pipeline for on-target and off-target determination is shown by the black arrow

Recently, we established an efficient CRISPR/Cas9 genome editing system in grapevine and obtained 22 *VvWRKY52* mutant plants from 72 T-DNA-inserted transgenic plants^12^. Here, we designed four CRISPR/Cas9 sgRNAs based on the sequence of *VvbZIP36*, a gene that has been shown to play a role in drought stress responses^47^ and obtained one mutant grapevine plant. The *Agrobacterium*-mediated genetic transformation process used is shown in Fig. 1. In our previous study, we analyzed six potential off-target sites from 12 transgenic *VvWRKY52* lines with biallelic mutations for off-targets using target sequencing. No off-target mutations were identified^12^, but the method used has a limited detection range. To comprehensively evaluate potential off-target effects on a genome-wide scale, we performed WGS of six *VvWRKY52* lines with biallelic mutations, as well as one *VvbZIP36* mutant and three WT (cv. Thompson Seedless) plants. The sequencing depth was approximately х58–х67. The sequencing depth of each independent line is listed in Table S3. For each gene, four sgRNAs were designed, as shown in Table S1. Three WT plants (control) were regenerated from embryogenic calli, and pro-embryonal masses (PEMs) were induced by floral explants (Fig. 1).

### WGS detection of on-target mutations

In our previous study, we tested on-target site mutations in four sgRNAs (sgRNA1, 2, 3, and 4) of Cas9-edited *VvWRKY52* lines by Sanger sequencing^12^. The results showed that the efficiency of gene editing was 28%, 6%, 17%, and 25% for the four sgRNAs. As a result, we obtained a total of 22 mutant plants from 72 T-DNA-containing transgenic plants^12^. After identifying the targeted mutations, we selected 6 lines (W52_37, 38, 42, 51, 52, and 60) with biallelic mutations for use in WGS. In this study, we also designed four sgRNAs (sgRNA5, 6, 7, and 8) for *VvbZIP36* (Table S1) and constructed a CRISPR/Cas9 multitarget vector, which was used for the transformation of Thompson Seedless plants. A total of 85 positive transgenic lines were obtained, of which only one mutant line (B36_45) was identified by Sanger sequencing (Fig. S1), and these lines were selected for WGS.

To select an appropriate reference genome for WGS analysis, the clean reads of 10 samples were mapped to the grape reference genome (PN40024; https://www.ncbi.nlm.nih.gov/) and Thomson Seedless genomes (http://openprairie.sdstate.edu/vitis_vinifera_sultanina/1). Compared with the Thomson Seedless (used for genetic transformation in this study) genome, the mapping rate on PN40024 as the reference genome was higher in all 10 samples (Table S4). One reasonable explanation is that the PN40024 reference genome is more complete than that of Thompson Seedless. Considering the better annotation of the PN40024 reference genome, it was used for the following analysis.

**Fig. 2:**
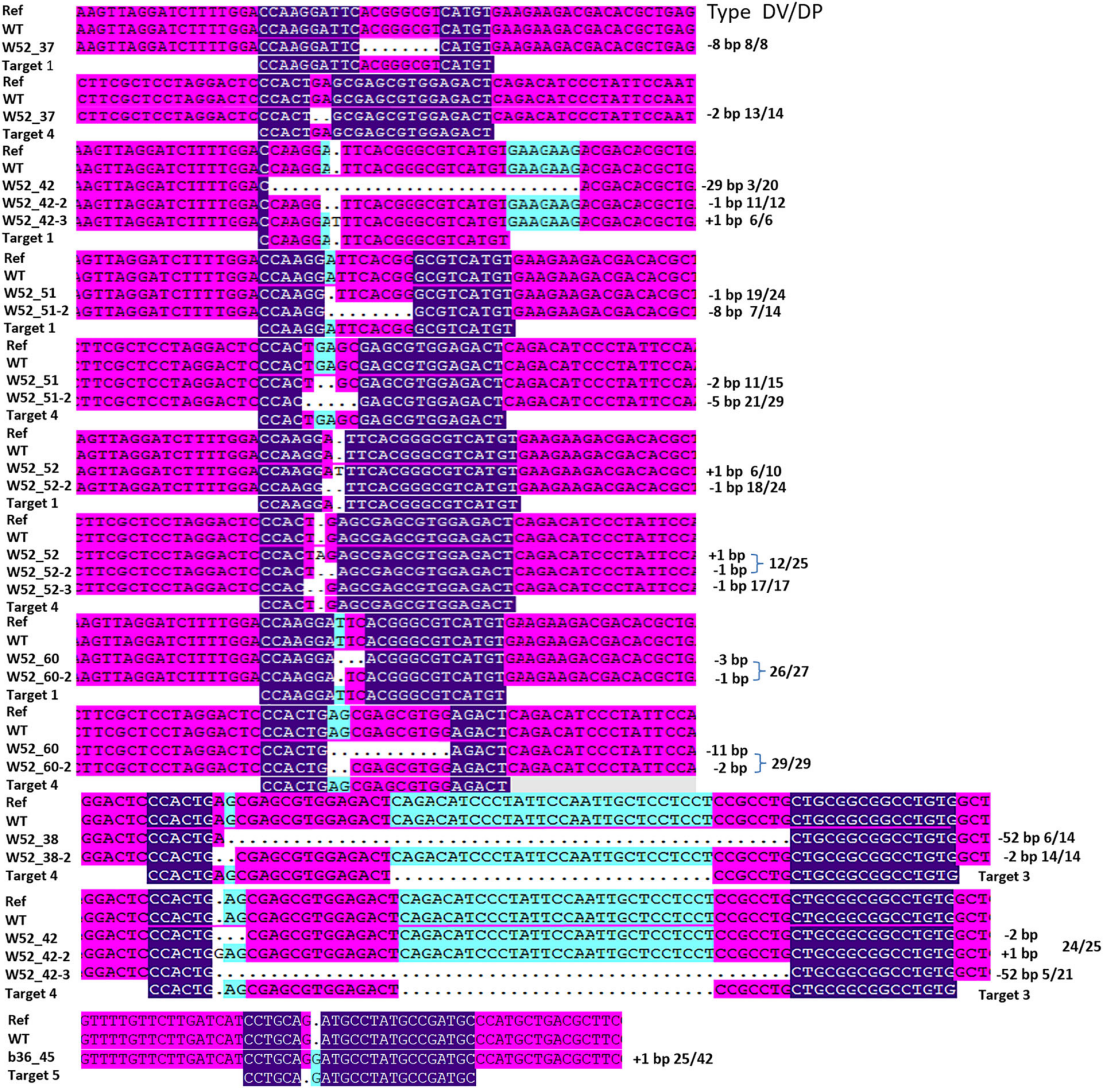
On-target analysis of seven Cas9-edited grapevine plants by whole-genome sequencing (WGS). ‘+’ represents insertion mutations, and ‘−’ represents deletion mutations. DV represents the number of reads of the variant base type at the site; DP represents the total number of reads covered by the site

The WGS data suggested that specific on-target mutations were introduced into CRISPR/Cas9-edited but not WT plants (Fig. 2). We found that three sgRNAs (sgRNA1, 3, and 4) induced on-target mutations in *VvWRKY52* and one sgRNA (sgRNA5) induced on-target mutations in *VvbZIP36* (Fig. 2). For sgRNA1, we detected five mutation types, including short insertions (+1), short deletions (−1, −3, and −8), and large deletions (−29). For sgRNA4, we detected five mutation types, including short insertions (+1) and short deletions (−1, −2, −5, and −11). For sgRNA5, only one short insertion (+1) was detected. In addition, a 52-bp deletion was detected in W52_38 and W52_52 between sgRNA3 and sgRNA4, consistent with the previous results^12^. These results indicated that different sgRNAs can induce different types of mutations and that the most common types of mutations are short insertions and deletions, indicating that the CRISPR/Cas9 system can be used for precise genome editing in the grapevine.

### SNP and indel analysis in WT and Cas9-edited plants

To identify potential off-target mutations, we analyzed the number of SNPs and indels in the 7 Cas9-edited plants. As shown in Table 1, compared to the grape reference genome, between 7,295,904 and 7,463,331 SNPs and between 617,915 and 639,742 indels were present as variants in the three WT plants. Most were genetic variations between Thompson Seedless, used in this study, and PN40024, used as the reference genome. A total of 6,551,278 SNPs and 513,774 indels were common to all three WT plants (Figs. S2, S3). In addition, compared to the grape reference genome, we identified between 7,308,740 and 7,724,670 SNPs and between 621,999 and 718,423 indels in the 7 Cas9-edited plants (Table 1). The variation between the Cas9-edited plants compared to the core variation, namely, the genetic variation shared by all three WT plants compared to the reference sequence of PN40024, was between 757,462 and 1,173,392 SNPs and between 108,225 and 204,649 indels (Table 1). We also identified between 202,008 and 272,397 SNPs and between 26,391 and 55,414 indels in the Cas9-edited transgenic grapevines that were not present in the three WT plants (Table 1 and Figs. S2, S3). For this reason, they were named “private variations”.

**Table 1:** Summary of total variations in wild-type (WT) and Cas9-edited plants

Description plants	Plants vs. Ref		Plants vs. WT		Private variations	
	Indel	SNP	Indel	SNP	Indel	SNP
WT_1	617915	7295904	–	–	–	–
WT_2	639742	7463331	–	–	–	–
WT_3	638472	7445663	–	–	–	–
B36_45	686411	7497964	172637	946686	49619	265170
W52_37	641658	7440800	127884	889522	29786	211379
W52_38	716760	7698041	202986	1146763	55020	264713
W52_42	621999	7308740	108225	757462	26391	202008
W52_51	718423	7724670	204649	1173392	55414	267238
W52_52	701625	7722911	187851	1171633	47673	272397
W52_60	654096	7542322	140322	991044	32711	232362

The ‘Plants vs. Ref’ column represents the variation (insertions/deletions (indels) and single nucleotide polymorphisms (SNPs) of each grapevine line compared to the reference genome (PN40024). ‘Plants vs. WT’ represents the variation of each Cas9-edited grape line compared to the core variations found in the three WT plants. The ‘Private variations’ indicate the variations only appearing in the Cas9-edited transgenic grapevine lines compared to the three WT plants

**Fig. 3:**
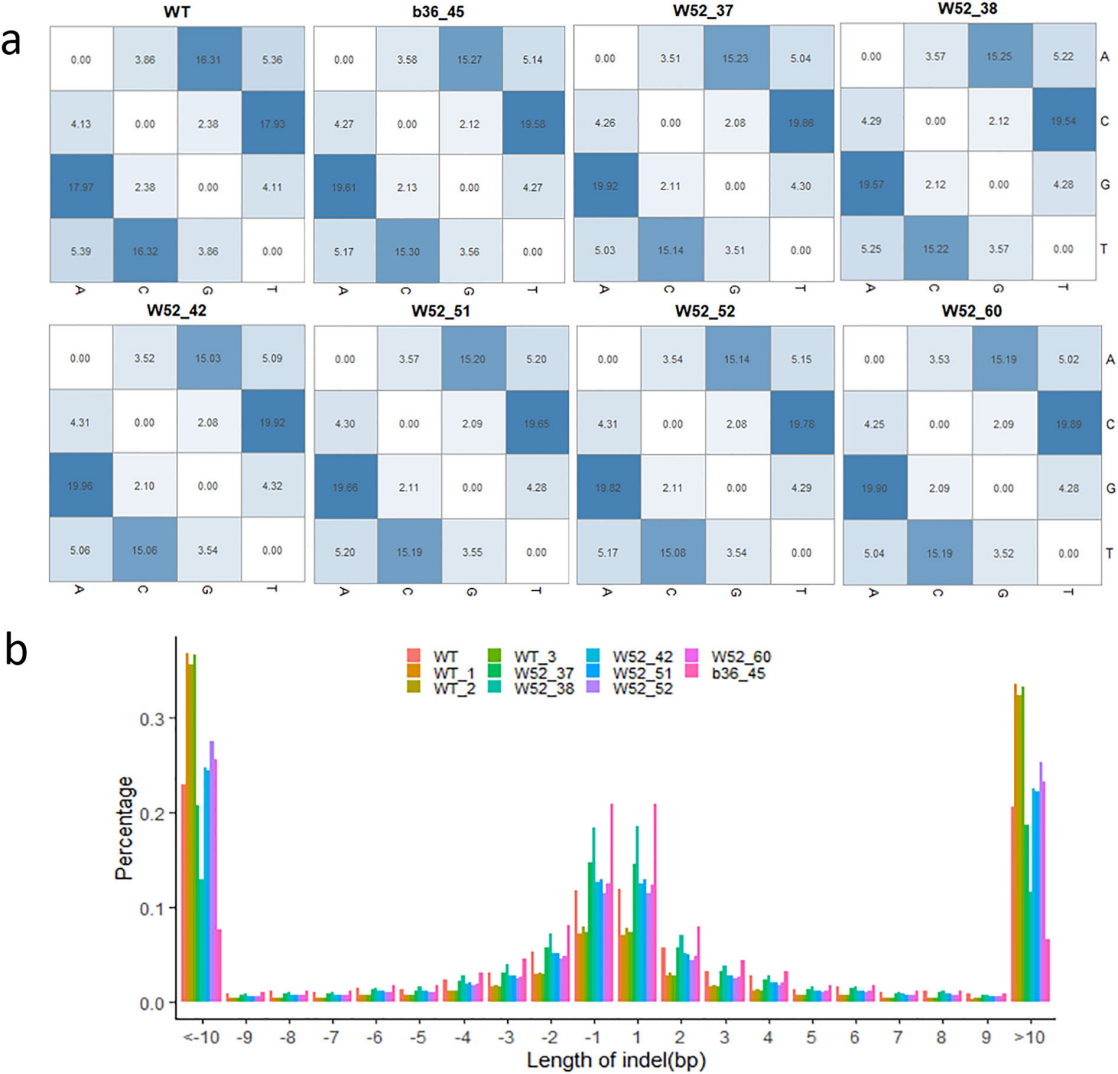
Genome-wide analysis of variations in Cas9-edited and wild-type (WT) plants regenerated from tissue culture. **a** Heat map representing the percentage of specific single nucleotide polymorphism (SNP) mutations in Cas9-edited and WT grapevine plants. **b** Analysis of indel length in WT and Cas9-edited plants

The annotation of these variations indicated that the fewest variations occurred in exon regions, and most variations occurred in intergenic regions (Table 2). We found between 27,224 and 35,927 SNPs and between 668 and 893 indels in exonic regions in the WT plants and between 36,549 and 47,086 SNPs and between 898 and 1270 indels in exonic regions in the Cas9-edited plants (Table 2). When analyzing the SNP mutation types, we found that A to G (15.03–15.27%), C to T (19.54–19.92%), G to A (19.57–19.92%), and T to C (15.06–15.30%) were the four most frequent mutations in the Cas9-edited plants (Fig. 3a). The most common indel variations were 1–2 bp in length, and these variations occurred more frequently in Cas9-edited plants than WT plants (Fig. 3b).

**Table 2:** Annotation of total variations in wild-type (WT) and Cas9-edited grapevine plants

Description plants	Exonic		Intronic		Upstream		Downstream		UTR		Intergenic	
	Indel	SNP	Indel	SNP	Indel	SNP	Indel	SNP	Indel	SNP	Indel	SNP
WT_1	668	27,224	18,984	106,351	9316	26,288	6370	20,442	2334	8203	65,553	553,874
WT_2	893	35,927	24,676	131,172	10,763	31,975	7649	25,846	3321	10,669	77,469	673,843
WT_3	865	35,007	24,402	127,809	10,682	31,968	7672	25,584	3217	10,300	76,659	661,121
B36_45	1206	43,252	38,013	167,370	16,884	44,503	11,882	34,803	5244	13,650	117,181	852,818
W52_37	1066	39,942	29,129	149,470	13,055	38,417	9161	30,139	3847	12,339	91849	767,933
W52_38	1221	45,021	40,857	173,586	18,775	47,654	12,822	37,005	5383	14,259	129,173	900,376
W52_42	898	36,549	25,971	139,409	11,939	36,515	8408	28,261	3407	11,435	85473	716,118
W52_51	1270	45,764	41,300	176,126	18,452	48,138	12,805	37,046	5525	14,429	130,575	916,485
W52_52	1190	47,086	37,820	175,747	17,111	47,295	11,919	36,318	4911	14,297	121,785	919,473
W52_60	1109	43,225	31,067	158,717	13,664	40,747	9490	31,829	4156	13,108	97421	820,100

### Off-target detection in Cas9-edited grapevine plants

**Fig. 4:**
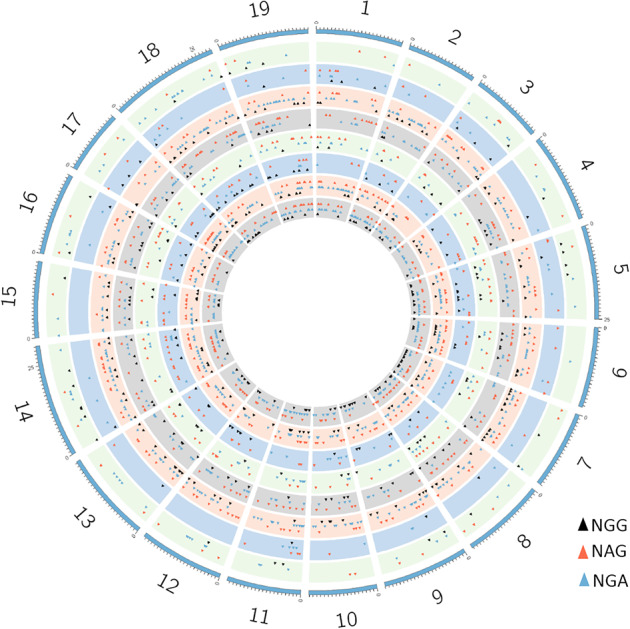
Genome-wide prediction of off-target sites using Cas-OFFinder. The numbers represent the 19 grape chromosomes, and the different colored triangles represent different types of potential off-target sites (NGG, NAG, NGA). Shown from the inside to the outside are the potential off-target sites of sgRNA1-8

**Fig. 5:**
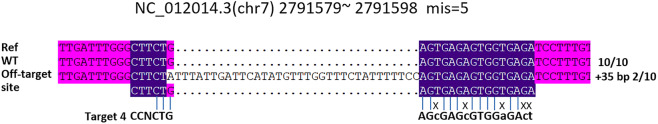
Identification of potential off-target mutations in Cas9-edited grapevine plants by Sanger sequencing. ‘Ref’ represents the reference genome sequence, and ‘WT’ represents the wild-type sequence. The number before the slash indicates the number of sequences of this type, and the number after the slash indicates the total number of sequences

To identify possible off-target mutations, the eight sgRNA sequences were aligned with the grape reference genome using Cas-OFFinder software^44^. Potential off-target sites with ≤5 mismatches in the sgRNAs were selected for further analysis. These comprised 603 (PAM: NGG), 939 (PAM: NAG), and 1730 (PAM: NGA) potential sites (Fig. 4, Fig. S4, Table 3 and Data S1). In the 7 Cas9-edited plants, we found only one indel variation in W52_52 (Table 3), which is likely due to the off-target activity of the Cas nuclease. Subsequently, Sanger sequencing was used to confirm this off-target mutation (Fig. 5). As reported previously, the types of mutations caused by the CRISPR/Cas9 system are often short insertions or short deletions^13^. Interestingly, the only off-target mutation we found was a 35-bp insertion (Fig. 5). These results suggest that the application of CRISPR/Cas9 to grapevine is highly specific and that few off-target mutations are generated.

**Table 3:** Whole-genome sequencing analysis of off-target events in Cas9-edited transgenic grapevine plants

Plants/sgRNA		Mutations/No. of NGG sites	Mutations/No. of NAG sites	Mutations/No. of NGA sites
W52-37	sgRNA1	0/32	0/40	0/83
	sgRNA2	0/21	0/36	0/37
	sgRNA3	0/134	0/139	0/295
	sgRNA4	0/75	0/138	0/168
W52-38	sgRNA1	0/32	0/40	0/83
	sgRNA2	0/21	0/36	0/37
	sgRNA3	0/134	0/139	0/295
	sgRNA4	0/75	0/138	0/168
W52-42	sgRNA1	0/32	0/40	0/83
	sgRNA2	0/21	0/36	0/37
	sgRNA3	0/134	0/139	0/295
	sgRNA4	0/75	0/138	0/168
W52-51	sgRNA1	0/32	0/40	0/83
	sgRNA2	0/21	0/36	0/37
	sgRNA3	0/134	0/139	0/295
	sgRNA4	0/75	0/138	0/168
W52-52	sgRNA1	0/32	0/40	0/83
	sgRNA2	0/21	0/36	0/37
	sgRNA3	0/134	0/139	0/295
	sgRNA4	0/75	1/138	0/168
W52-60	sgRNA1	0/32	0/40	0/83
	sgRNA2	0/21	0/36	0/37
	sgRNA3	0/134	0/139	0/295
	sgRNA4	0/75	0/138	0/168
B36-45	sgRNA5	0/49	0/100	0/90
	sgRNA6	0/91	0/84	0/51
	sgRNA7	0/102	0/181	0/782
	sgRNA8	0/99	0/221	0/224

The eight sgRNA sequences were aligned to the grape reference genome (PN40024) using Cas-OFFinder. All potential off-target areas with ≤5 mismatches were selected for subsequent analysis

### Analysis of new off-targets generated by genetic variation among Thompson Seedless and the grape reference genome, PN40024

Considerable genomic variation between the Thompson Seedless cultivar, which is often used for grapevine transformation, and the reference cultivar (PN40024) was identified. Considering that the analysis of potential off-target sites is based on the grape reference genome, such variations might affect the interpretation of the results of this study. To take this into account, we used the 6,551,278 SNPs and 513,774 indels overlapping in the three WT plants to “correct” the grape reference genome (Figs. S2, S3), and the newly generated reference genome was used for potential off-target mutation analysis. This resulted in the identification of 47 (PAM: NGG), 60 (PAM: NAG), and 136 (PAM: NGA) new potential off-target sites (Table 4 and Data S2). When we analyzed the variation in these new potential off-target sites, no mutations marking off-target events were identified based on the WGS data.

**Table 4:** New potential off-target sites in the “corrected” reference genome sequence by genetic variations in the wild-type (WT) plants

Target sgRNA	Off-target sites (NGG)	Off-target sites (NAG)	Off-target sites (NGA)
sgRNA1	0/1	0/7	0/3
sgRNA2	0/3	0/2	0/0
sgRNA3	0/4	0/3	0/12
sgRNA4	0/5	0/11	0/18
sgRNA5	0/4	0/7	0/3
sgRNA6	0/7	0/4	0/4
sgRNA7	0/18	0/13	0/81
sgRNA8	0/5	0/13	0/15

The numbers in front of the ‘/’ symbol represent the off-target variations in Cas9-edited grapevine according to whole-genome sequencing, and the numbers after the ‘/’ symbol represent new potential off-target sites

## Discussion

In recent years, the CRISPR/Cas9 system has been successfully applied to edit target genes in grapevine^12,14,30–33,48^. For example, Ren et al.^48^ showed that the *IdnDH* gene can be edited in ‘Chardonnay’ suspension cells^48^, and we reported the editing of a transcription factor, *VvWRKY52*, in the Thompson Seedless cultivar^12^. Sunitha et al. produced transgenic plants with CRISPR/Cas9 targeting *TAS4b* and *MYBA7* in the 101-14 rootstock and obtained 2 independently edited *TAS4b* lines and 5 edited *MYBA7* lines^33^. Moreover, Wan et al. showed that CRISPR/Cas9 *VvMLO3*-edited grape lines had enhanced resistance to grapevine powdery mildew^32^, and Li et al. reported that *VvPR4b* loss-of-function lines had decreased resistance to *P. viticola*^30^.

Early studies in human cells found high frequencies of off-target mutations in CRISPR/Cas9-edited cells^18,19^, Understanding the reason or mechanism of off-target mutations is crucial for the application of this technology, particularly for medical applications^13,19^. Previous studies in grapevine claimed that no off-target events occurred in Cas9-edited plants^30,31,48^; however, these studies relied on in silico prediction of potential off-target sites and verification by target sequencing, which is somewhat biased and limited in scope. In this study, we performed a large-scale WGS analysis of three WT and 7 Cas9-edited plants targeting two genes (*VvWRKY52* and *VvbZIP36*) to detect potential off-target sites caused by the use of eight Cas9 sgRNAs.

The WGS analysis detected up to ~7.7 million SNPs and ~718,000 indels in the 7 Cas9-edited plants and as many as ~7.5 million SNPs and ~64,000 indels in the 3 WT plants compared to the reference genome sequence of PN40024 (Table 1). The large genomic variation observed within WT plants or Cas9-edited plants has three main sources: (i) heterozygosity of grapevines; (ii) tissue culture-induced mutations; and (iii) pre-existing/inherent mutations between different grape cultivars. As shown in Figs. S2 and S3, most of the mutations were shared by WT plants and Cas9-edited transgenic grapevines. Referring to the results of the previous off-target analysis in cotton^13^, these mutations are mainly pre-existing/inherent mutations between different grape cultivars. In addition, most of the Cas9-edited lines had more variations than WT plants (Table 1). One explanation for this is that expanded tissue culture and/or *Agrobacterium* infection of Cas9-edited plants may induce mutations, as has been shown in the previous studies^17^.

The annotation of these variations showed that between 27,224 and 35,927 SNPs and between 668 and 893 indels in exonic regions were present in the WT plants and between 36,549 and 47,086 SNPs and between 898 and 1270 indels in exonic regions were present in the Cas9-edited plants (Table 2). The Cas9-edited lines had more variations in exonic regions than the WT plants. In view of the relatively small variations caused by on-target and off-target effects (Fig. 2 and Fig. 5), these variations were mainly produced by tissue culturing and/or *Agrobacterium* infection. Similar results have been reported in rice^17^ and cotton^13^. These spontaneous mutations that occur in the exonic regions might affect the function of those genes, possibly interfering with the phenotypic analysis of Cas9-edited plants. This could be a problem for applying CRISPR/Cas9 to functional genomics research. However, selecting multiple independent regenerated mutants for phenotypic analysis can effectively solve this problem.

The on-target analysis of the 7 Cas9-edited lines by WGS revealed that short insertions and short deletions were the most common types of mutations (Fig. 2), consistent with our previous results^12^ and similar studies in cotton^13^ and maize^15^. We also found a 52-bp deletion in W52_38 and W52_52 between sgRNA3 and sgRNA4 in our WGS data, consistent with the Sanger sequencing results of our previous study^12^. These results underline the high reliability of WGS data for detecting on-target and/or off-target mutations.

To identify off-target mutations with higher precision, we first predicted the potential off-target sites of the eight sgRNAs using the grape reference genome (PN40024); second, we predicted the potential off-target sites in a “corrected” genome sequence, taking into account the different cultivars used for the gene-editing experiment. Many computational tools have been developed for off-target analysis, including CasOT^49^, OffScan^49^, and Cas-OFFinder^44^; however, they were originally developed to detect off-targets in animals^13^. Of these, Cas-OFFinder is most often used for off-target prediction in plants, as described for cotton^13^ and rice^17^, and for this reason, we used this software in this study. We identified 603 (PAM: NGG), 939 (PAM: NAG), and 1730 (PAM: NGA) potential off-target sites with ≤ 5 mismatches in the sgRNAs (Fig. 4, Table 3, Table S5 and Data S1). sgRNA1 and 2 were predicted to have a lower potential for off-target mutations, and sgRNA7 had the highest potential (Table S5), which highlights the importance of sgRNA design to ensure specificity in genome editing.

Next, we analyzed all the predicted SNPs and indel variations in the potential off-target sites of the eight sgRNAs but found only one actual indel variation by using sgRNA4 in W52_52 (Table 3 and Fig. 5). This is indicative of the very low off-target mutation rate due to Cas9 genome editing in the grapevine. Similarly, low rates or no off-targets have been found in other plant species, such as rice, where Zhang et al.^21^ did not detect any off-target mutations among multiple CRISPR/Cas9-edited lines by WGS^21^. Similar results have been reported in maize^15^ and *A. thaliana*^16^. In cotton, four bona fide off-target indel mutations were detected by WGS^13^. This higher rate may be due to the more complex and larger genome (2.5 Gb) compared to grapevine (430 Mb) or the target design. The only off-target mutation detected in the 7 Cas9-edited grapevine lines in this study was a 35-bp insertion, while in cotton, all four off-target mutations were short deletions^13^, suggesting randomness in the DSB repair process induced by Cas9.

The risk of off-target mutations is increased in plants where the CRISPR/Cas9 construct is active for a long time^50^. Some researchers are committed to developing a way to obtain clean edited plants to reduce such off-target risks^50,51^. Six of the Cas9-edited grapevines of W52 used in this study were obtained in our previous studies^12^. These Cas9-edited lines with the CRISPR/Cas9 construct have experienced approximately 30 months of growth from regeneration to off-target analysis. However, only one off-target indel mutation was identified. These results suggest that the long-term existence of the Cas9 construct in grapevines does not cause a large number of off-target mutations. In view of this finding, it is not urgent to obtain clean edited plants without Cas9 and gRNA integration on grapevines to reduce off-target risks. In addition, these observations imply that Cas9-induced mutagenesis is highly specific in grapevines.

The current standard for off-target analysis relies on using a reference genome, such as Col-0 for *A. thaliana*, Nipponbare for rice, TM-1 for cotton, and PN40024 for grapevine^12,13^. However, the cultivars/genotypes used for reference genome sequencing are often not widely used for genetic transformation. In this study, we found ~6.6 million SNPs and ~513,000 indels in the Thompson Seedless cultivar used for gene editing compared with the reference genome (PN40024) (Table 1). These variations affect accuracy when predicting potential off-target sites, so a “correction” of the grape reference genome sequence was performed based on overlapping variations in the three WT plants (Figs. S2, S3) prior to the second round of potential off-target prediction. This resulted in 47 (PAM: NGG), 60 (PAM: NAG), and 136 (PAM: NGA) predicted off-target sites, but no off-target mutations were detected by sequencing at any of these sites (Table 4 and Data S2). Taken together, these results indicate that the CRISPR/Cas9 system is highly specific in grapevine, and compared with variations caused by tissue culturing and/or *Agrobacterium* infection, the off-target mutations caused by Cas9 are likely insignificant.

## Supplementary information

Supplementary Figures

Table S1-S5

Dataset 1

Dataset 2
