# Supplementary material for: Whole-genome sequencing reveals rare off-target mutations in CRISPR/Cas9-edited grapevine
Source: Hortic Res. 2021 May 1;8:114. doi: 10.1038/s41438-021-00549-4 (PMC8087786; doi:10.1038/s41438-021-00549-4)
Supplement: Supplementary file 1 — Supplementary Figures [file 41438_2021_549_MOESM1_ESM.doc]

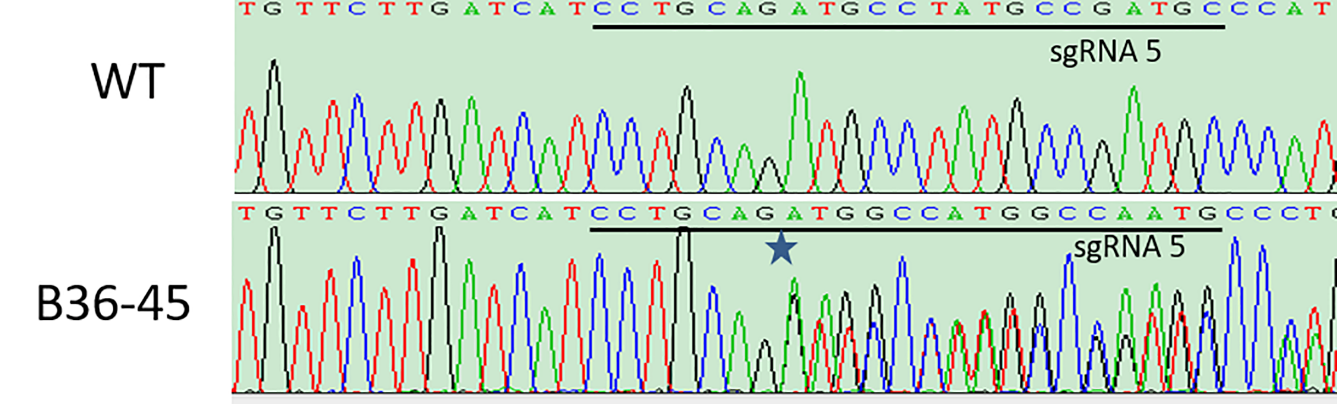


**Fig. S1** On-target mutation analysis of *VvbZIP36* Cas9-edited grapevine plants by Sanger sequencing.


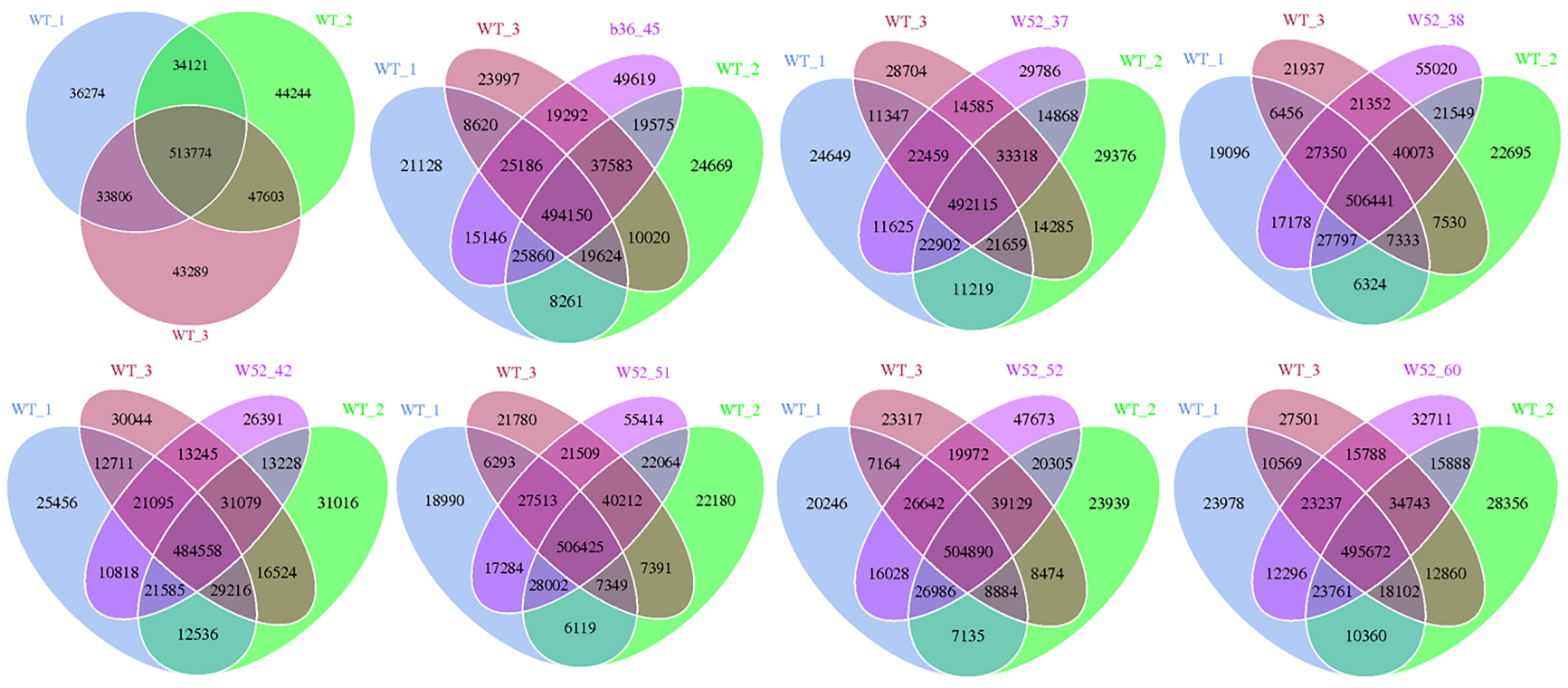


**Fig. S2** Unique and common indels in the seven *VvWRKY52* and *VvbZIP36* Cas9-edited grapevine lines.


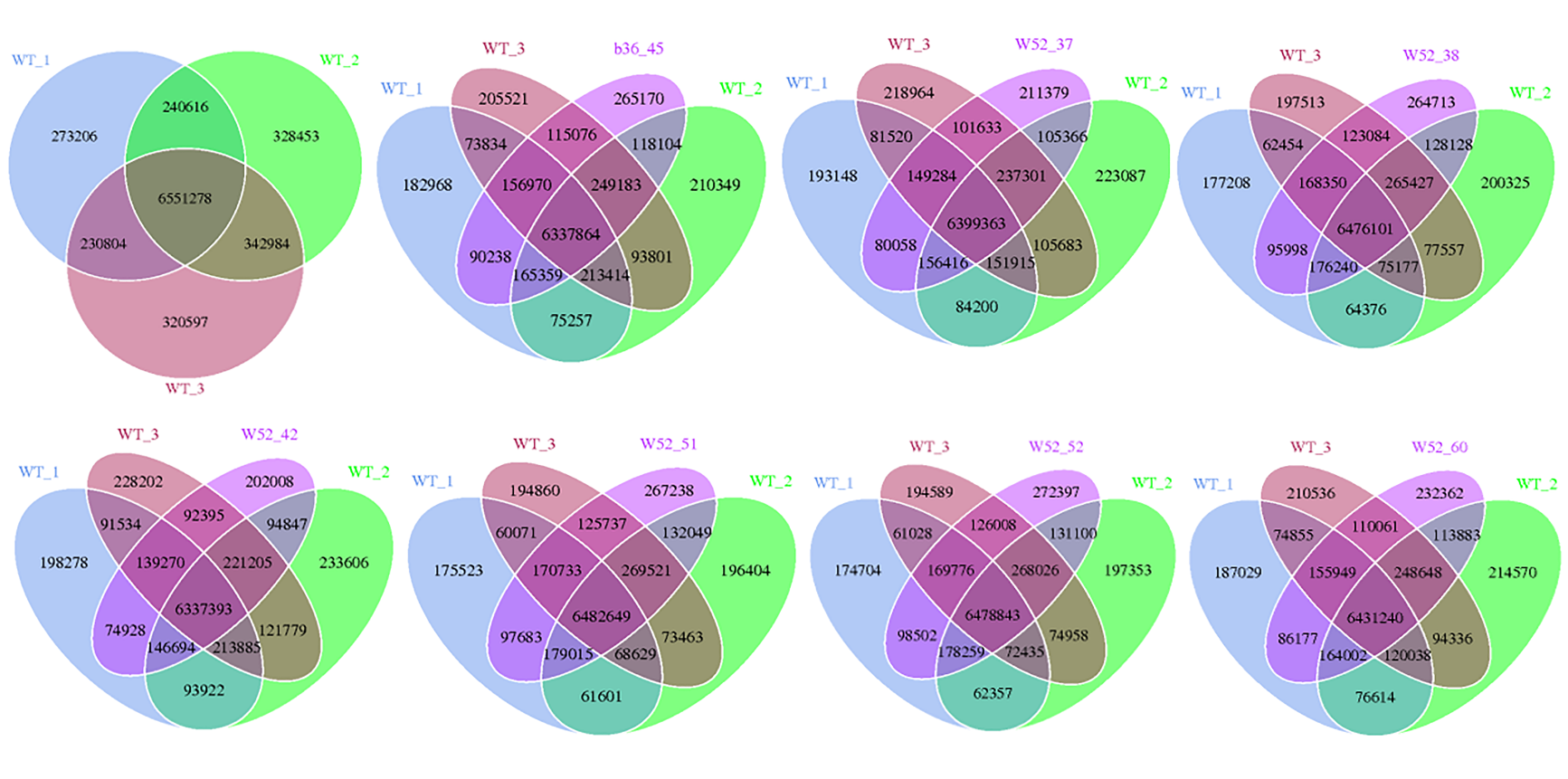


**Fig. S3** Unique and common single nucleotide polymorphisms (SNPs) in seven *VvWRKY52* and *VvbZIP36* Cas9-edited grapevine lines.


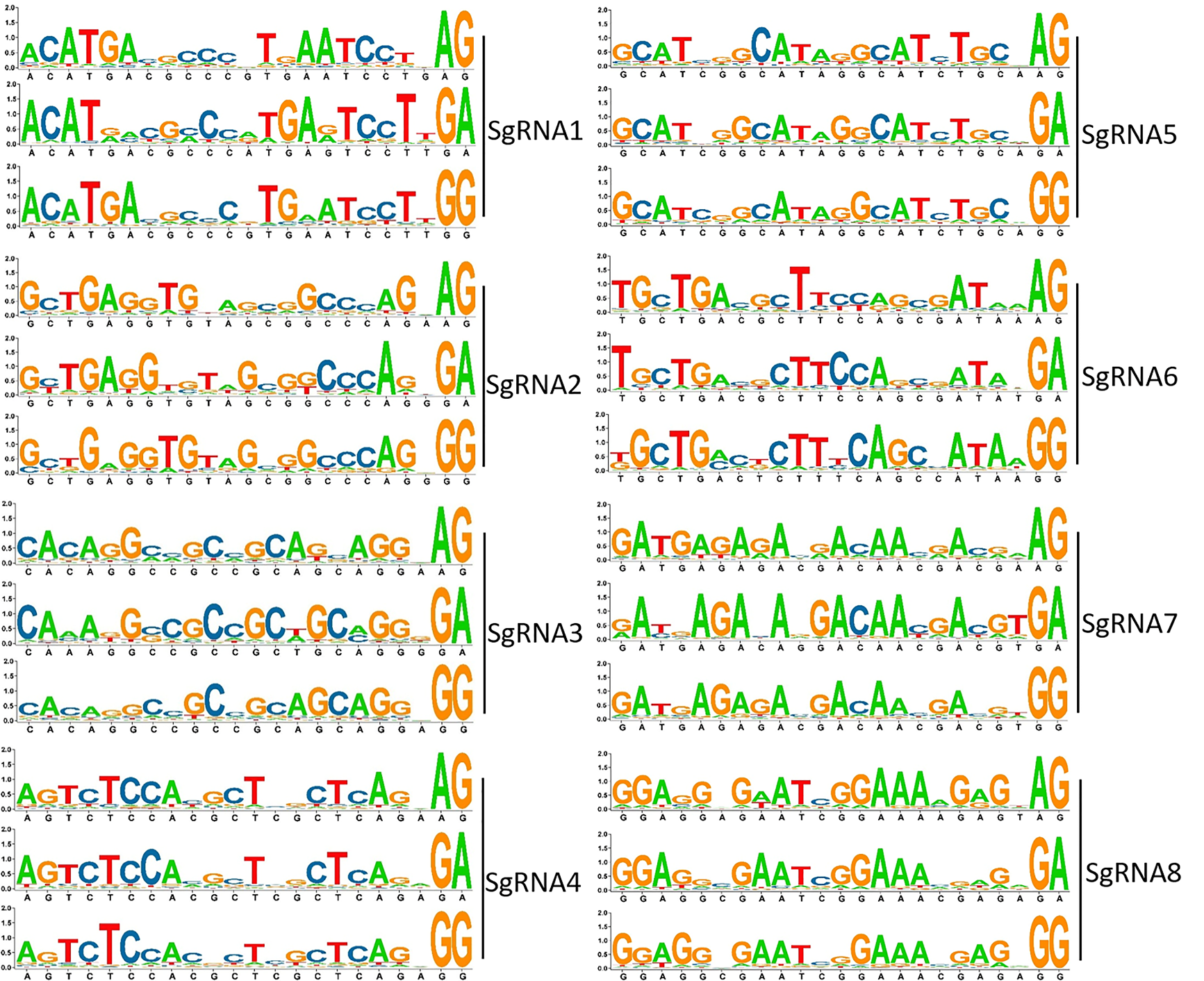


**Fig. S4** Logos for potential off-target sequences.
