# Supplementary material for: Whole-genome sequencing reveals rare off-target mutations in CRISPR/Cas9-edited grapevine
Source: Hortic Res. 2021 May 1;8:114. doi: 10.1038/s41438-021-00549-4 (PMC8087786; doi:10.1038/s41438-021-00549-4)
Supplement: Supplementary file 2 — Table S1-S5 [file 41438_2021_549_MOESM2_ESM.doc]

**Table S1** Information of eight sgRNAs used for CRISPR/Cas9 editing

| **SgRNA** | **Sequence** | **Strand** | **Start** | **Chr** |
| --- | --- | --- | --- | --- |
| **SgRNA1** | ACATGACGCCCGTGAATCCTTGG | - | 19479417 | NC_012022.3 |
| **SgRNA2** | GCTGAGGTGTAGCGGCCCAGTGG | - | 19479354 | NC_012022.3 |
| **SgRNA3** | CACAGGCCGCCGCAGCAGGCGG | - | 19479326 | NC_012022.3 |
| **SgRNA4** | AGTCTCCACGCTCGCTCAGTGG | - | 19479275 | NC_012022.3 |
| **SgRNA5** | GCATCGGCATAGGCATCTGCAGG | - | 420968 | NC_012024.3 |
| **SgRNA6** | TGCTGACGCTTCCAGCGATAAGG | + | 420994 | NC_012024.3 |
| **SgRNA7** | GATGAGAGACGACAACGACGAGG | + | 420883 | NC_012024.3 |
| **SgRNA8** | GGAGGtGAATCGGAAACGAGGGG | - | 420794 | NC_012024.3 |

**Table S2** Primers used for vector construction and off-target analysis

| gRB36T1+ | GCATCGGCATAGGCATCTGCgttttagagctagaaat-3’ |
| --- | --- |
| gRB36T2+ | TGCTGACGCTTCCAGCGATAgttttagagctagaaat-3’ |
| gRB36T3+ | GATGAGAGACGACAACGACGgttttagagctagaaat-3 |
| gRB36T4+ | GGAGGCGAATCGGAAACGAGgttttagagctagaaat-3 |
| AtU3bB36T1- | GCAGATGCCTATGCCGATGC**T**gaccaatgttgctcc-3’ |
| AtU3dB36T2- | TATCGCTGGAAGCGTCAGCA**C**aatcactacttcgtct-3’ |
| AtU3bB36T3- | CGTCGTTGTCGTCTCTCAT**C**aatctcttagtcgact-3’ |
| AtU3dB36T4- | CTCGTTTCCGATTCGCCTCC**T**gaccaatggtgctttg-3’ |
| F-off | CTTCCTCCACACCTCGCCA |
| R-off | AATCCTTTAGGAAAGATGATTTCTTCC |

**Table S3** Depth of the whole genome sequencing data of wild type and Cas9-edited grapevine lines

| **Plants** | **Clean reads** | **Depth** |
| --- | --- | --- |
| W52_37 | 205548368 | 61 |
| W52_38 | 202122292 | 60 |
| W52_42 | 198131514 | 58 |
| W52_51 | 228956322 | 67 |
| W52_52 | 218245624 | 65 |
| W52_60 | 199927492 | 59 |
| WT_1 | 208887270 | 61 |
| WT_2 | 203823862 | 60 |
| WT_3 | 205213244 | 60 |
| b36_45 | 217078934 | 61 |

**Table S4** Statistical analysis of average sequencing depth and map rate

| **sample** | **Thompson Seedless genomes** | | | **PN40024** | | |
| --- | --- | --- | --- | --- | --- | --- |
|  | avg_depth | map_rate | total_map_base | avg_depth | map_rate | total_map_base |
| W52_37 | 55 | 81.42 | 25104895730 | 61 | 83.37 | 25173423301 |
| W52_38 | 54 | 89.76 | 27215071349 | 60 | 92.00 | 27372475769 |
| W52_42 | 53 | 83.11 | 24700137300 | 58 | 84.60 | 24624426943 |
| W52_51 | 61 | 83.77 | 28770188620 | 67 | 84.59 | 28478936476 |
| W52_52 | 59 | 84.74 | 27741892800 | 65 | 85.91 | 27563757847 |
| W52_60 | 54 | 79.82 | 23938388136 | 59 | 81.35 | 23884905867 |
| WT_1 | 56 | 83.10 | 26037006887 | 61 | 85.34 | 26190905682 |
| WT_2 | 54 | 80.08 | 24483703229 | 60 | 82.38 | 24662632334 |
| WT_3 | 55 | 78.92 | 24293719812 | 60 | 80.79 | 24357564894 |
| B36_45 | 55 | 81.80 | 26635719994 | 61 | 86.87 | 27807452707 |

**Table S5** Number of potential off-target sites of eight sgRNAs

| **SgRNA** | **NGG** | **NAG** | **NGA** | **Total** |
| --- | --- | --- | --- | --- |
| **SgRNA1** | 32 | 40 | 83 | 155 |
| **SgRNA2** | 21 | 36 | 37 | 94 |
| **SgRNA3** | 134 | 139 | 195 | 568 |
| **SgRNA4** | 75 | 138 | 168 | 381 |
| **SgRNA5** | 49 | 100 | 90 | 239 |
| **SgRNA6** | 91 | 84 | 51 | 226 |
| **SgRNA7** | 102 | 181 | 782 | 1065 |
| **SgRNA8** | 99 | 221 | 224 | 544 |
| **Total** | 603 | 939 | 1730 | - |
